# The development of L-type Ca^2+^ current mediated alternans does not depend on the restitution slope in canine ventricular myocardium

**DOI:** 10.1038/s41598-021-95299-7

**Published:** 2021-08-17

**Authors:** Noémi Tóth, Jozefina Szlovák, Zsófia Kohajda, Gergő Bitay, Roland Veress, Balázs Horváth, Julius Gy. Papp, András Varró, Norbert Nagy

**Affiliations:** 1 Department of Pharmacology and Pharmacotherapy, Faculty of Medicine, University of Szeged, Dóm tér 12, P.O. Box 427, 6720 Szeged, Hungary; 2 ELKH-SZTE Research Group of Cardiovascular Pharmacology, Szeged, Hungary; 3 Department of Physiology, Faculty of Medicine, University of Debrecen, Debrecen, Hungary; 4 Faculty of Pharmacy, University of Debrecen, Debrecen, Hungary; 5 Department of Pharmacology and Pharmacotherapy, Interdisciplinary Excellence Centre, University of Szeged, Szeged, Hungary

**Keywords:** Cardiology, Arrhythmias

## Abstract

Cardiac alternans have crucial importance in the onset of ventricular fibrillation. The early explanation for alternans development was the voltage-driven mechanism, where the action potential (AP) restitution steepness was considered as crucial determining factor. Recent results suggest that restitution slope is an inadequate predictor for alternans development, but several studies still claim the role of membrane potential as underlying mechanism of alternans. These controversial data indicate that the relationship of restitution and alternans development is not completely understood. APs were measured by conventional microelectrode technique from canine right ventricular papillary muscles. Ionic currents combined with fluorescent measurements were recorded by patch-clamp technique. APs combined with fluorescent measurements were monitored by sharp microelectrodes. Rapid pacing evoked restitution-independent AP duration (APD) alternans. When non-alternating AP voltage command was used, Ca^2+^_i_-transient (CaT) alternans were not observed. When alternating rectangular voltage pulses were applied, CaT alternans were proportional to I_CaL_ amplitude alternans. Selective I_CaL_ inhibition did not influence the fast phase of APD restitution. In this study we found that I_CaL_ has minor contribution in shaping the fast phase of restitution curve suggesting that I_CaL_—if it plays important role in the alternans mechanism—could be an additional factor that attenuates the reliability of APD restitution slope to predict alternans.

## Introduction

Cardiac alternans refer to a regular beat-to-beat oscillation of the ECG T-waves caused by parallel alternans of the AP and the CaT at the cellular level^1,2^. It is generally accepted that alternans are associated with arrhythmogenesis and were identified as a suitable predictor of sudden cardiac death^3,4^. Large body of evidence demonstrated that cardiac alternans are complex phenomenon, where mutual crosstalk of the membrane potential changes and alterations of the intracellular Ca^2+^ play a key role in the development of alternans (for review^5–7^). At the same time, it is uncertain whether the AP or the intracellular Ca^2+^ initialize and govern alternans.

Regarding the underlying mechanism of alternans, two major concepts have been emerged: voltage-driven and Ca^2+^-driven alternans. Growing body of recent evidence indicate the importance of Ca^2+^ handling in the development of alternans. *Ca*^*2*+^*-driven alternans* were originally suggested to develop as a result of release-reuptake mismatch following steep dependence of sarcoplasmic reticulum (SR) release on loading^7,8^, and later the refractory state of the ryanodine receptor was assumed as the underlying mechanism^9^. Third possibility is the refractoriness of the Ca^2+^ cycling proteins that arises from the combination of steep load-release relationship, and refractoriness of the SR release^10^. Recent evidence indicates the ‘3R-theory’ that claims the alternans rises via an instability caused by interactions between three critical properties: randomness of Ca^2+^ sparks, recruitment of Ca^2+^ sparks by neighboring Ca^2+^ release units (CRU), and refractoriness of CRUs^11,12^. An important milestone of the Ca^2+^-driven hypothesis was several reports demonstrating that CaT alternans can be evoked in the presence of non-alternating AP sequence^13–16^.

The concept of *voltage-driven alternans* represents an early explanation for the underlying mechanism of alternans development. A general view is that the voltage-driven alternans are governed by the restitution slope, i.e. alternans are expected to develop when the slope of AP duration-diastolic interval (APD-DI) function is larger than 1^17^. This means that restitution-hypothesis—which was originally proposed by Nolasco and Dahlen^17^—represents an ultimate underlying factor of the voltage-driven alternans^1,5,7^.

The restitution hypothesis was challenged by several laboratories^1,18^, at the same time there are experimental and modeling papers claiming important role of membrane voltage in the development of alternans^19–22^. This discrepancy suggests that the relationship of restitution and alternans is not completely understood and requires further investigations.

## Methods

### Ethical statement

All experiments were conducted in compliance with the *Guide for the Care and Use of Laboratory Animals* (USA NIH publication No 85-23, revised 1996) and conformed to Directive 2010/63/EU of the European Parliament. The protocols were approved by the Review Board of the Department of Animal Health and Food Control of the Ministry of Agriculture and Rural Development, Hungary (XIII./1211/2012). Animal studies were carried out in compliance of ARRIVE guidelines.

### Determination of the action potential parameters in canine multicellular papillary muscle

Beagle dogs (obtained from a licensed supplier, licence number: XXXV./2018) from both sex weighing 10–15 kg were used for conventional microelectrode experiments. The animals were anesthetized and sacrificed with pentobarbital (60 mg/kg iv), then the heart was removed through a right lateral thoracotomy.

APs were recorded at 37 °C from the surface of right ventricular papillary muscles using conventional microelectrode technique. For measuring tissue APs similar protocol was applied as described earlier with minor modifications^23,24^. Briefly, the preparations were mounted in a custom made Plexiglas chamber, allowing continuous super-fusion with Locke’s solution (130 mM NaCl, 21.5 mM NaHCO_3_, 4.5 mM KCl, 12 mM glucose, 0.4 mM MgCl_2_, 1.8 mM CaCl_2_, ph 7.35 ± 0.05) and stimulated with constant pulses of 5 ms duration at a rate of 1 Hz through a pair of bipolar platinum electrodes using an electro-stimulator (EX-ST-A2, Experimetria Ltd). Sharp microelectrodes with tip resistance of 10–20 MΩ, when filled with 3 M KCl, were connected to an amplifier (Biologic Amplifier, model VF 102). Voltage output from the amplifier was sampled using an AD converter (NI 6025, Unisip Ltd). APs were monitored and evaluated by using Evokewave v1.49 (Unisip Ltd).

#### APD alternans protocol

APD alternans were measured by rapid pacing using cycle lengths of 250-230-210-190-160 ms. 20 consecutive stimuli were used at each frequency. APD differences were calculated between long and short AP pairs along at least 6 consecutive pulses at 25, 50 and 90% of repolarization (APD_25,_ APD_50_ and APD_90_, respectively) and were averaged providing APD alternans amplitude.

#### Conventional S1S2 restitution protocol

The basic cycle length (BCL) was 1000 and 500 ms. Extra delays for S2 stimuli ranged from − 50 to 1000 ms related to the baseline APD_90_. 15 consecutive stimuli were applied (S1) between all S2 stimuli. Diastolic intervals (DI) refer to the proximity of S2 stimuli (extra beat) to the APD_90_ of the basic beat evoked by S1.

#### Dynamic restitution protocol

Following a pre-pacing at 1 Hz, the basic cycle length was gradually decreased from 1000 to 500 ms by 100 ms steps, from 500 to 300 ms by 50 ms steps. When the cycle length reached the 250 ms the action potential started to alternate and the AP alternation was constantly maintained from 250 to 160 ms. In this cycle length interval, we applied the previously described cycle length pattern for alternans. During these measurements the APD_90_ was plotted against the preceding diastolic interval at each cycle length.

Previous study reported that standard S1S2 restitution method is not suitable tool to predict alternans since the slope in most cases < 1^25^. However, we observed restitution slopes larger and smaller than 1 in approximately 50–50% of our experiments, providing an average value of 1.12 ± 0.1 (BCL: 1000 ms; n = 20) and 1.08 ± 0.1 (BCL: 500 ms; n = 20). Therefore, we considered standard S1S2 restitution protocol as an appropriate tool to assess restitution slope.

### Voltage-clamp measurements combined with fluorescent recordings

The isolation of canine left ventricular cardiomyocytes of Beagle dogs’ hearts was performed as described previously^26^. In brief, cardiac myocytes were isolated from the left ventricle, containing an arterial branch through which the segment was perfused on a Langendorff apparatus with solutions in the following sequence: normal isolation solution (containing in mM: 135 mM NaCl, 4.7 mM KCl, 1.2 mM KH_2_PO_4_, 1.2 mM MgSO_4_, 1 mM CaCl_2_, 10 mM Glucose, 10 mM HEPES, 20 mM taurine, 4.4 mM NaHCO_3_, 5 mM Na-pyruvic acid, pH 7.2 adjusted with NaOH) for 10 min, Ca^2+^-free isolation solution for 10 min and isolation solution containing collagenase (Worthington type II, 0.66 mg/mL) and 33 µM CaCl_2_. To the final perfusion solution protease (type XIV, 0.12 mg/mL) was added at the 15th minutes for digestion. The isolated cardiomyocytes were loaded with Fluo-4-AM (1–2 μM, Molecular Probes, USA; AM is the membrane permeable acetoxymethyl ester conjugated form of the dye) for 20 min at room temperature in dark. The loaded cells were placed in a low volume imaging chamber (RC47FSLP, Warner Instruments, USA), and the cells were then continuously perfused with normal Tyrode solution at 37 °C (1 mL/min). Data acquisition and analysis were performed using Axon Digidata 1550B System (Molecular Devices, Sunnyvale, CA, USA). Parallel with the current measurements, the fluorescent recordings were performed on a stage of an inverted fluorescent microscope (IX71, Olympus, Japan) and the signal was recorded by a photomultiplier module (H7828, Hamamatsu, Japan), sampled at 1 kHz. Data acquisition and analysis were (both current and Ca^2+^ transient) performed using the pClamp 11.0 software (Molecular Devices, Sunnyvale, CA, USA).

Calcium alternans were based on six consecutive CaTs, where the average CaT amplitude was computed for even and odd beats. The amplitude of a single CaT was estimated as the difference between the peak and the minimum directly preceding the first analyzed CaT (i.e., it is the CaT upstroke amplitude).

### Parallel measurement of AP and CaT with current clamp technique

Single cell AP measurements were performed as described previously^27^. Rod-shaped viable ventricular cardiomyocytes, showing clear striation, were placed in a 1 ml volume experimental chamber mounted on the stage of an inverted microscope (Nikon Diaphot 300; Nikon Co., Tokyo, Japan). After sedimentation, cardiomyocytes were continuously superfused with at 37 °C Tyrode solution at a rate of 1–2 mL/min. Cells were impaled with 3 M KCl filled conventional borosilicate microelectrodes having tip resistances between 20 and 40 MΩ, connected to the input of a Multiclamp 700A amplifier (Molecular Devices, Sunnyvale, CA, USA). Action potentials (AP) were elicited through these intracellular electrodes by applying 2 ms wide rectangular current pulses having amplitudes of twice the diastolic threshold. The membrane potential signal was digitalized at 50 kHz using Digidata 1440A A/D card, recorded with pClamp 10 software (both from Molecular Devices) and stored for later analysis. APs were recorded at pacing cycle lengths of 1000, 500, 300, 250, 230, 210 ms in this respective order. Cardiomyocytes were loaded with 5 µM Fura-2 AM for 30 min at room temperature in a Pluronic F-127 containing Tyrode solution. 25 mg Pluronic F-127 was dissolved in 1 mL DMSO and this solvent was used to make a Fura-2 AM stock solution of 1 mM. After loading, the cells were washed twice with Tyrode solution, and were allowed to rest for 30 min at room temperature to de-esterify the dye, and then they were stored at 15 °C before the experiments. Fluorescence was measured using an alternating dual beam excitation fluorescence photometry setup (RatioMaster; Photon Technology International, New Brunswick, NJ, USA) coupled to the inverted microscope. Fluorescence signals of Ca^2+^-bound and Ca^2+^-free Fura-2 dye were detected at excitation wavelengths of 340 nm (F_340_) and 380 nm (F_380_), respectively. Emitted photons were detected at 510 nm with an R1527P photomultiplier tube (Hamamatsu Photonics K.K., Hamamatsu, Japan). This signal was digitalized at 200 Hz using the FelixGX software (Photon Technology International) and stored for offline analysis. Background fluorescence was measured by moving the cell out of the field of view, and it was subtracted from total fluorescence in order to obtain fluorescence originating exclusively from the preparation. Fluorescence ratio of F_340_/F_380_ was used to assess intracellular Ca^2+^ transients (CaT). The CaT was recorded in parallel with AP and CL.

### Measurement of I_CaL_

The L-type calcium current (I_CaL_) was recorded in HEPES-buffered Tyrode’s solution, supplemented with 3 mM 4-aminopyridine under perforated patch (Fig. 2) or whole-cell configuration (Fig. 3).

To obtain *I*_*CaL*_* current and alternans* (Fig. 3), 3 separated voltage protocols were used. In all protocols, the current was activated by voltage steps to 0 mV after a 25 ms long prepulse at − 40 mV. The voltage protocols differed in the length of consecutive voltage pulses used to evoke I_CaL_: (1) 140/80 ms (2) 180/40 ms (3) 200/20 ms. The holding potential was − 80 mV, the basic cycle length was 220 ms in all cases. The cells were loaded with Fluo-4-AM fluorescent dye, and the experiments were performed by perforated patch technique with a pipette solution containing (in mM): 120 K-gluconate, 2.5 NaCl, 2.5 MgATP, 2.5 Na_2_ATP, 5 HEPES, 20 KCl, titrated to pH 7.2 with KOH. During perforated patch-clamp experiments 50 µM β-escin was added to the pipette solution. I_CaL_ current measurements with buffered Ca^2+^_i_ (10 mM EGTA) were performed with whole cell configuration with pipette solution containing (in mM): 125 CsCl, 20 TEACl, 5 MgATP, 10 HEPES, 10 NaCl titrated to 7.2 with CsOH.

*Recovery characteristic* of I_CaL_ was obtained by activation of the I_CaL_ current by rectangular pulses from a holding potential of − 80 mV to 0 mV for 400 ms. Subsequently interpulse intervals were used from 25 to 500 ms before the second rectangular pulse that was identical with the initial pulse. The I_CaL_ recovery was calculated by comparing the initial I_CaL_ peaks evoked by the initial pulse with I_CaL_ pulse elicited by the second pulse, and were plotted against the corresponding interpulse intervals. During these experiments the Ca^2+^ handling was intact. The cells were loaded with Fluo-4-AM fluorescent dye, and experiments were performed by whole-cell patch-clamp technique.

All chemicals were purchased from Sigma (USA). All experiments were carried out at 37 °C.

### Statistics

Normal distribution of the data was verified by using Shapiro–Wilk test. Statistical significance (p < 0.05) was assessed using Student’s t-test. Data are presented as mean ± S.E.M.

## Results

### Action potential alternans and restitution measurements in intact subendocardial right ventricular papillary muscle

**Figure 1:**
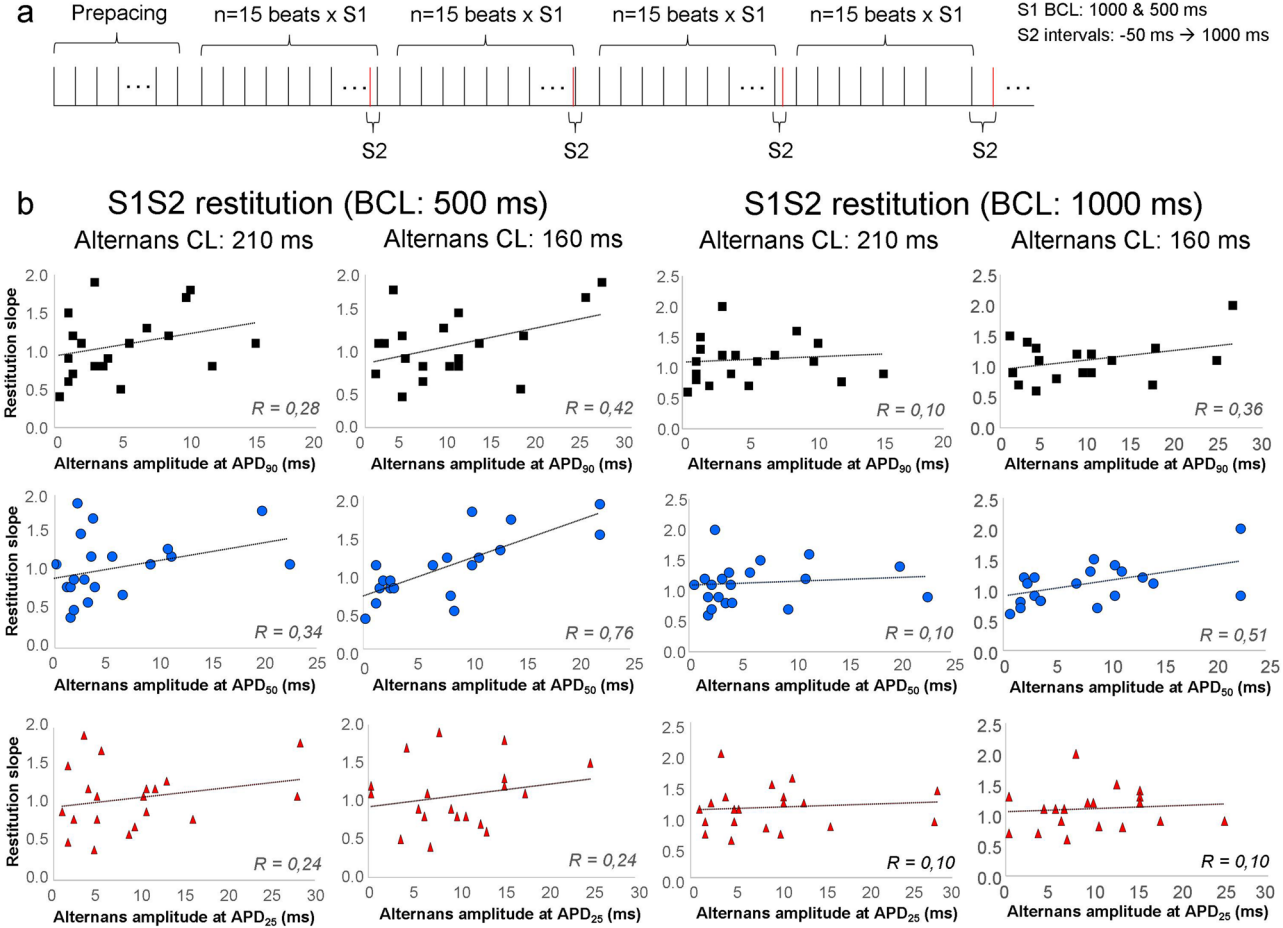
Relationship between action potential alternans and S1S2 restitution. **(a)** Illustrates the applied protocol for S1S2 restitution. The basic cycle length for restitution was 1000 and 500 ms. The diastolic intervals increased from − 50 to 1000 ms (relative to APD_90_) and the slope of APD_90_ restitution was calculated at both 500 and 1000 ms BCLs. After the restitution protocols, alternans were measured from a cycle length of 250 ms to 160 ms. The magnitude of alternans was measured at APD_90_, APD_50_, and APD_25_. **(b)** Demonstrates correlation tests between S1S2 restitution slope and alternans. The left 6 panels show S1S2 restitutions at BCL of 500 ms, in the right 6 panels the BCL was 1000 ms. The first row demonstrates alternans at APD_90_ level, middle row at APD_50_, bottom row at APD_25_. Our results indicate that alternans developed even if the restitution slope was smaller than 1, furthermore, data show mainly weak and in some cases moderate correlations between variables.

In the first set of experiments APD restitution (S1S2 protocol) and APD alternans protocol were measured and compared from the same intact subendocardial tissue (right ventricular papillary muscle). Figure 1 represents the summary of 20 experiments (from 11 dogs) of control S1S2 restitution—alternans experiments. Figure 1a illustrates the applied S1S2 restitution protocol. The S1S2 restitution was recorded at BCL of 1000 then 500 ms. The APD alternans amplitude was defined from at least 6 consecutive, regular short-long APs at APD_90_, APD_50_ and APD_25_ as APD difference. Alternans could be evoked in all cases (n = 20) with clear threshold, i.e. when the pacing length became equal or shorter than 250 ms (action potential waveforms can be seen in Supplementary Fig. S1). When AP alternans developed, they were maintained during the entire pacing protocol without any decline in the magnitude of APD oscillation.

Figure 1b represents comparisons between restitution slopes and the corresponding magnitude of APD alternans. Left side of the panel represents S1S2 restitutions at BCL of 500 ms, right side illustrates restitutions at 1000 ms (restitution was analyzed at APD_90_ level). Alternans measured at 210 ms cycle length (odd columns) and 160 ms (even columns) were highlighted. The first row represents alternans amplitudes measured at APD_90_ level, middle row shows alternans recorded at APD_50_ level, and bottom row illustrates alternans at 25% of repolarization. Our results indicate that alternans developed even if the restitution slope was smaller than 1, furthermore, data show mainly weak and in some cases moderate correlations between variables.

We also analyzed the S1S2 restitutions at APD_50_ and APD_25_ and these values were plotted against the corresponding alternans magnitude. Weak or moderate correlations were found (Supplementary Fig. S2).

**Figure 2:**
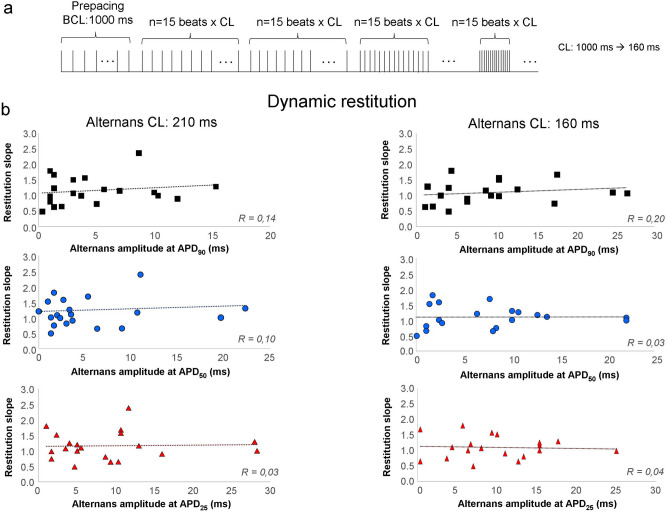
Relationship between action potential alternans and dynamic restitution. **(a)** Illustrates the applied protocol for dynamic restitution. The basic cycle length for restitution was progressively decreased from 1000 to 160 ms. When BCL was 250 ms or shorter the action potential started to alternate. APD_90_ was plotted against the preceding diastolic interval in both long and short AP during alternans. **(b)** The slope of APD_90_ restitution was calculated and was plotted against the magnitude of alternans. The first row demonstrates alternans at APD_90_ level, middle row at APD_50_, bottom row at APD_25_. Similarly to the S1S2 restitution, we found that alternans developed even if the slope was smaller than 1, and data exerted weak or no correlations between restitution slope and alternans.

Figure 2 demonstrates comparisons of restitution slopes obtained from dynamic restitution and alternans amplitude. Figure 2a depicts the applied dynamic restitution protocol: the pacing cycle length was gradually decreased. At cycle length of 250 ms the APD started to alternate.

Functions of restitution slope—alternans amplitude are illustrated in Fig. 2b. Left column represents alternans at cycle length of 210 ms, right column represents the same at cycle length of 160 ms. The first row shows alternans amplitudes measured at APD_90_ level, middle row represents alternans recorded at APD_50_ level, and bottom row illustrates alternans at 25% of repolarization. Similarly to the S1S2 restitution, we found that alternans developed even if the slope was smaller than 1, and data exerted weak or no correlations between restitution slope and alternans.

The temporal relationship of alternans and restitution slopes was further illustrated in Supplementary Fig. S3. It can be observable that alternans could be evoked at slower pacing cycle lengths (i.e. 250 ms) where the corresponding restitution curves were slow, indicating that alternans can be evoked even if the restitution slope is smaller than 1 (Supplementary Fig. S3).

### Non-alternating AP sequence failed to elicit CaT alternans

**Figure 3:**
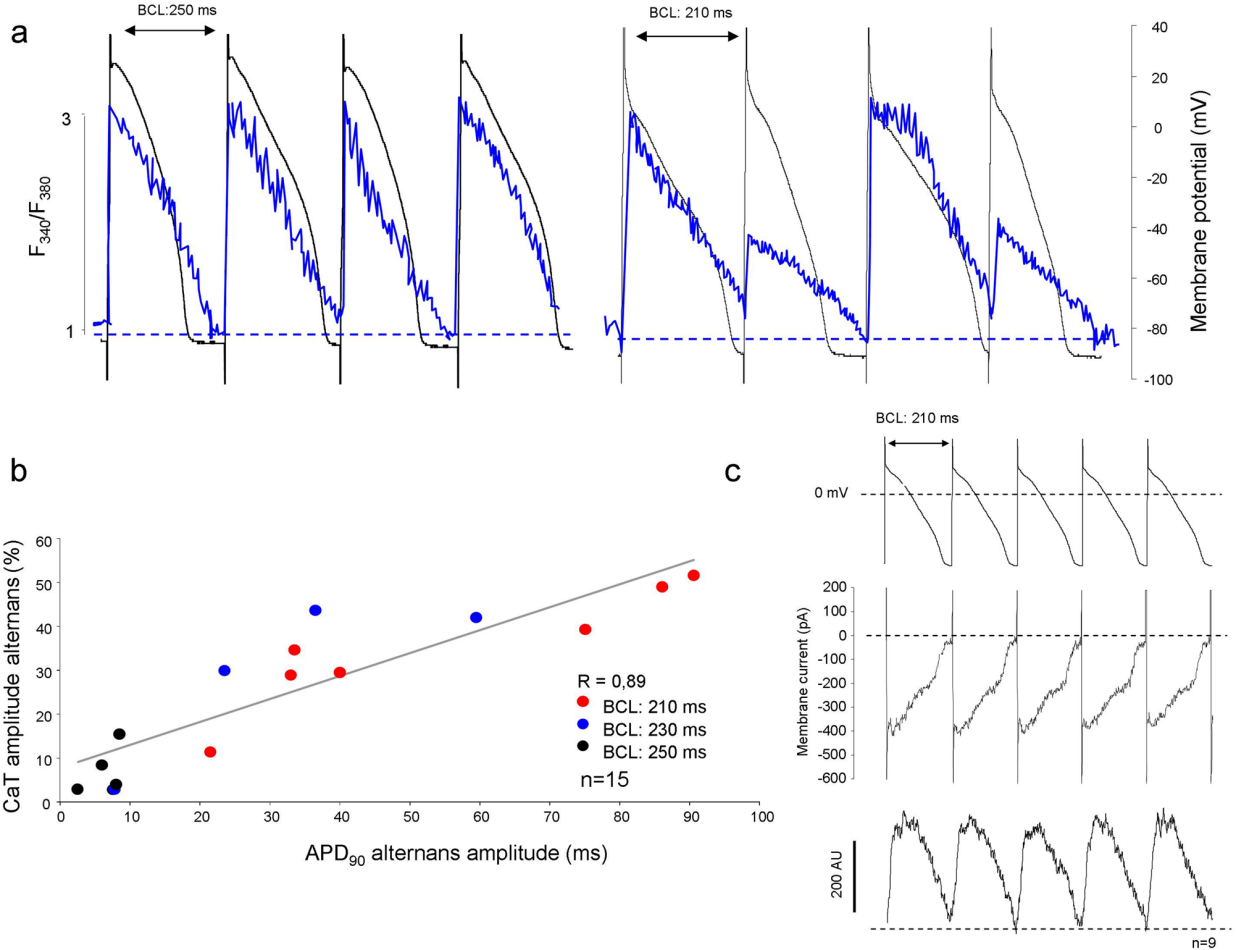
Relationship between APD and CaT amplitude alternans. **(a)** Illustrates simultaneous measurements of AP and CaT recorded at 250, 230 and 210 ms of BCL. At the left side a moderate AP alternans (black) coupled with marginal alternans of the CaT (blue curve), measured at 250 ms. **(a)** Right side illustrates marked AP alternans (black) associated with pronounced CaT alternans (blue) monitored at 210 ms. **(b)** Illustrates the APD_90_ alternans amplitude plotted against the extent of CaT alternans defined as CaT peak alternans ratio, at various BCLs. The grey line represents the linear regression of the points. **(c)** Depicts rapid pacing by non-alternating membrane potential, (upper curve) failed to induce CaT alternans under perforated patch-clamp condition. Parallel measured membrane current (middle curve) and CaT (lower curve) traces at a basic cycle length of 210 ms are shown. The evoked membrane currents were identical, and the evoked CaTs exerted negligible alternans.

In order to further address the relationship between AP and CaT alternans, current clamp experiments by using sharp microelectrodes were performed in single cells (Fig. 3a). Stimulus pattern from a cycle length of 250 to 210 ms was applied. The CaT amplitude oscillations were plotted against the corresponding APD_90_ difference (Fig. 3b) within the range of 250–210 ms stimulus cycle length. A close relationship between APD and CaT amplitude alternans was found: larger APD alternans were associated with larger CaT amplitude alternans (n = 15).

In order to elucidate the initiator mechanism of alternans, a non-alternating AP sequence having a BCL of 210 ms was applied under voltage-clamp mode (Fig. 3c upper panel). During measurements, the ionic currents (Fig. 3c middle panel) and CaT (Fig. 3c lower panel) were monitored. The experiments were performed by perforated patch-clamp method to preserve the intracellular milieu. Here we found that the inward ionic currents (other currents were inhibited, see Methods) as well as CaT exerted (6.7 ± 2.2%, n = 9) negligible alternans. These results suggest that in our experiments the action potential alternans are required for CaT alternans.

### I_CaL_ kinetics during alternating voltage pulses

Among transmembrane ionic currents I_CaL_ could be the most obvious candidate that may have important role in the alternans mechanism. As a trigger of the Ca^2+^ release it can directly influence the magnitude of the actual Ca^2+^ transient, and its kinetic is strongly influenced by the membrane potential.

**Figure 4:**
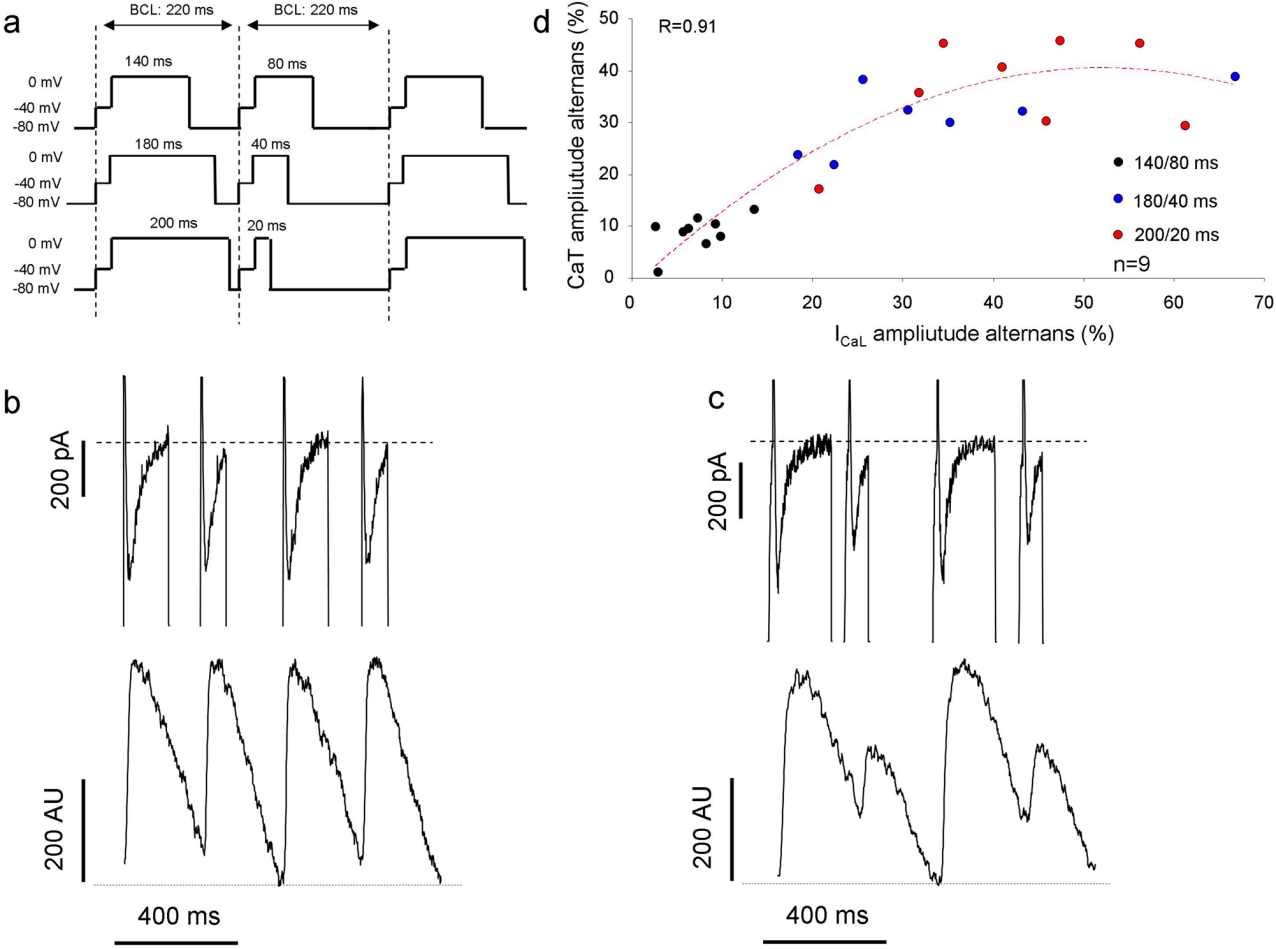
The role of I_CaL_ alternans in ignition of CaT alternans. I_CaL_ were evoked by 3 subsequently applied alternating voltage clamp pulses, having a basic cycle length of 220 ms in all cases **(a)**. **(b)** Illustrates a membrane current and CaT measurement evoked by a 140/80 ms voltage protocol. The I_CaL_ peaks as well as the corresponding CaT did not show notable alternans. **(c)** Represents alternating I_CaL_ and CaT during application of 180/40 ms protocol. **(d)** Illustrates a close relationship when the peak I_CaL_ alternans were plotted against the corresponding CaT amplitude alternans. The black circles represent experiments evoked by 140/180 ms protocol, the blue circles demonstrate measurements under 180/40 ms protocol, while red circles indicates recordings during 200/20 protocol.

In order to address the contribution of I_CaL_ in alternans, we applied 3 alternating voltage pulse protocols to produce different extent of I_CaL_ alternans due to alternans of the recovery time (Fig. 4a). The measurements were performed by using perforated patch clamp method. The magnitude of corresponding CaT amplitude alternans was analyzed and compared to the I_CaL_ amplitude alternans. The BCL of all protocols was 220 ms in all cases (see description in the “Methods” section).

In these experiments a close correlation between I_CaL_ amplitude alternans and corresponding CaT amplitude alternans was found (Fig. 4d). It implies that CaT alternans were marginal when I_CaL_ alternans were small even though the high pacing frequency. Thus, these results are in agreement with the observed failure of the high pacing rate to produce alternans (Fig. 3c) and the close relationship between APD and CaT alternans demonstrated in Fig. 3a,b.

The Ca^2+^ release channel recovery is also sensitive to membrane potential oscillations^9^. Therefore, there is a possibility that an alternating Ca^2+^ release controlled by ryanodine recovery could also directly contribute to the observed I_CaL_ alternans. In order to assess this issue, whole cell patch clamp experiments were devised where 10 mM EGTA was employed to buffer Ca^2+^_i_ (Supplementary Fig. S4). In the absence of Ca^2+^ release the average of I_CaL_ alternans did not differ from those that were measured in the presence of intact Ca^2+^ handling (presented in Fig. 4) indicating major role of membrane potential in the development of I_CaL_ alternans (140 ms—EGTA-free: 7.3 ± 1.2% *vs* EGTA: 6.5 ± 1.3%. 180 ms—EGTA-free: 25.7 ± 6% *vs* EGTA: 21.3 + 3%. 200 ms—EGTA-free: 41.4 + 4% *vs* EGTA: 45.5 ± 7%. n(EGTA-free) = 9, n(EGTA) = 6)).

### Recovery of the I_CaL_

**Figure 5:**
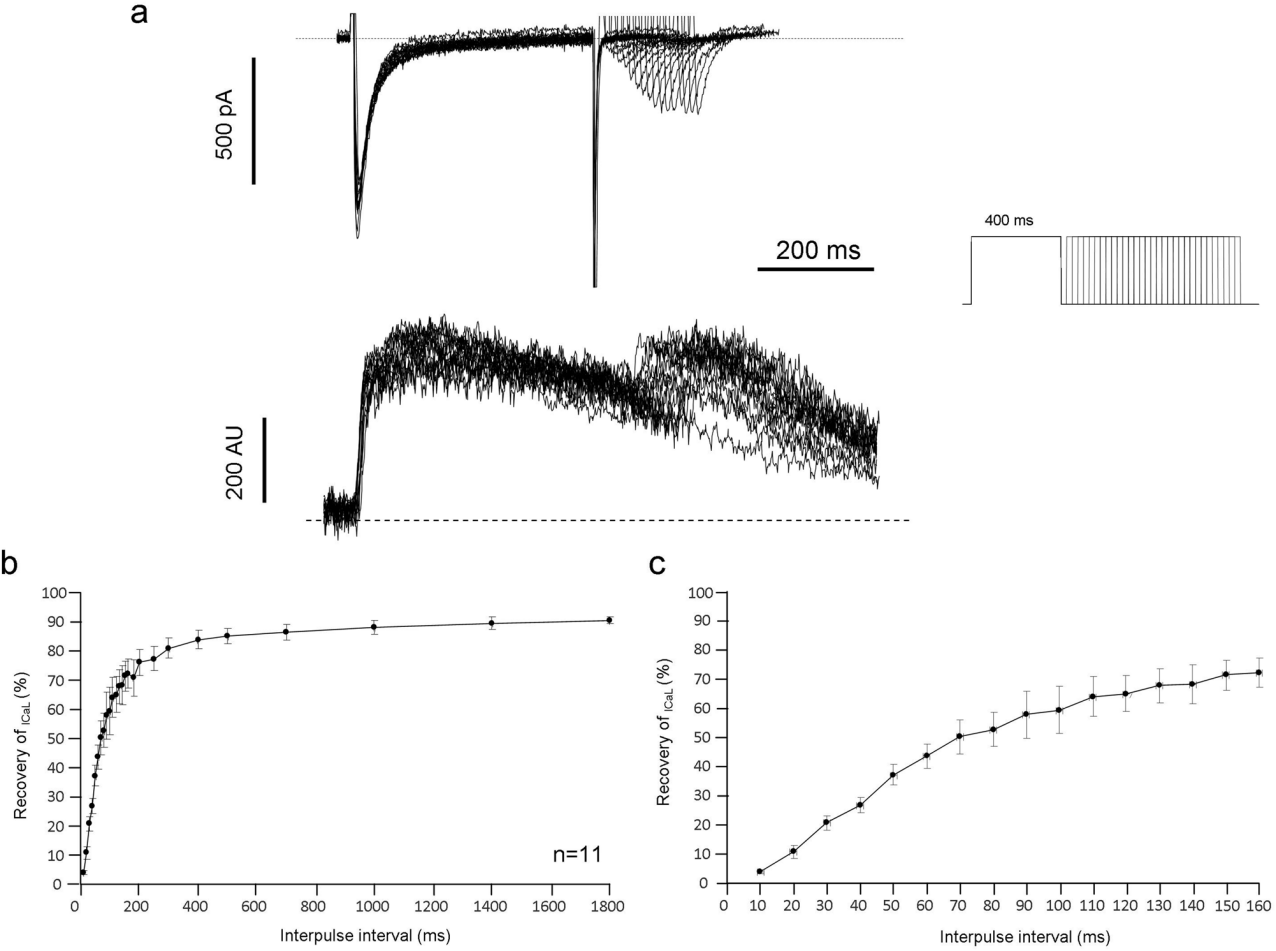
Analysis of the I_CaL_ recovery in the presence of unbuffered Ca^2+^ handling. **(a)** (upper traces) demonstrates original representative current traces of recovery kinetics of the I_CaL,_ Inset shows the applied voltage protocol. The lower panel demonstrates the parallel Ca^2+^ releases. The **(b)** depicts the extent of I_CaL_ recovery plotted against the respective diastolic intervals, while **(c)** highlights the first 160 ms of the I_CaL_ recovery.

A further important question is the behavior of I_CaL_ during the fast phase of the restitution curve. This issue was investigated by whole-cell patch clamp omitting Ca^2+^ buffer from the pipette solution (Fig. 5). The description of the recovery protocol can be found in the Methods section. We found that during the first 30 ms of the DI, where the APD restitution is the fastest, the I_CaL_ recovers only 20.7 ± 2.4% (n = 11) providing relatively small (0.6 ± 0.08 pA/pF, n = 11) current (Fig. 5b,c). These results indicate that I_CaL_ has restricted contribution to the initial, rapid phase of restitution.

### I_CaL_ inhibition suppresses alternans without influencing the initial phase of restitution

**Figure 6:**
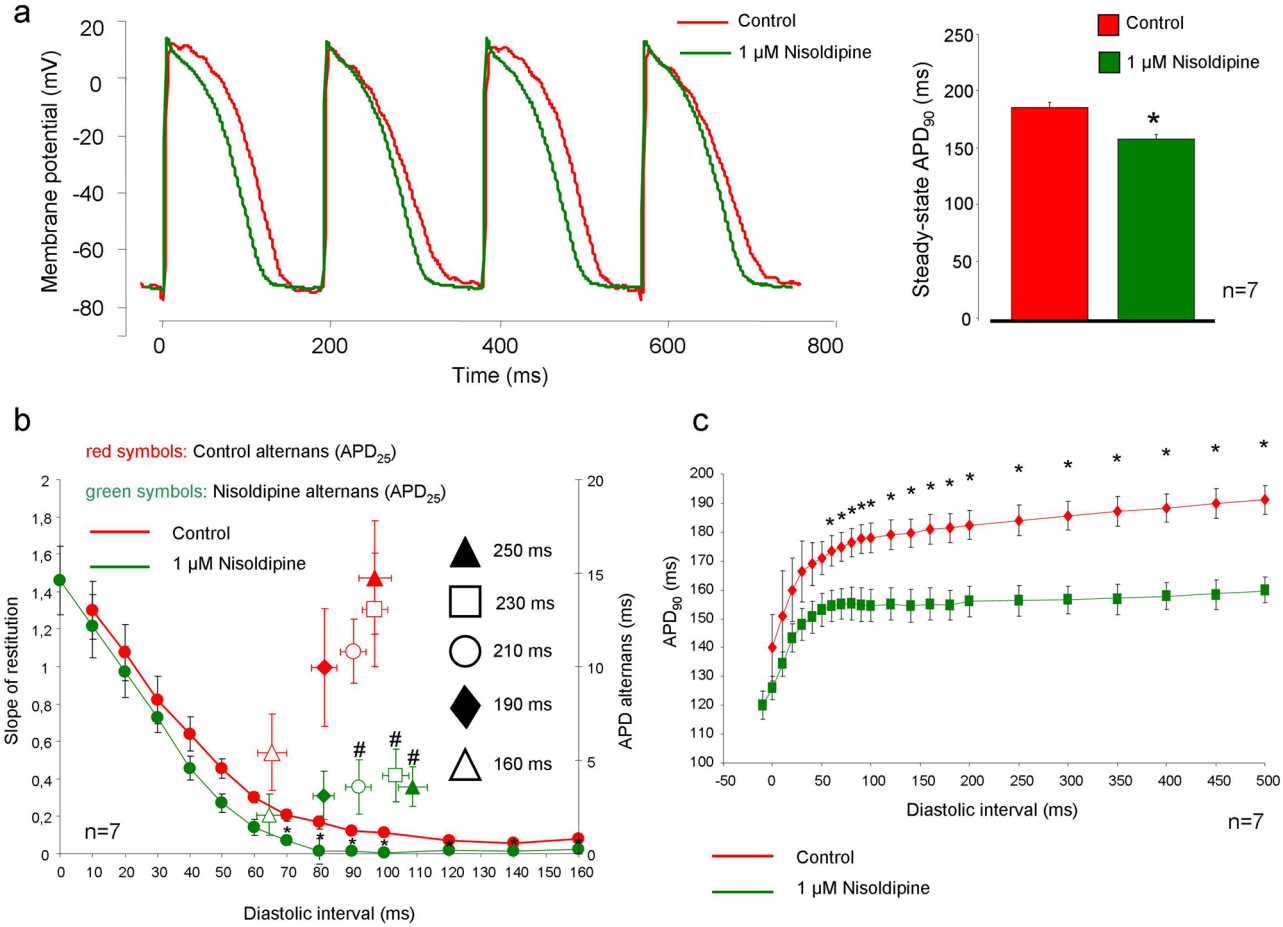
Effect of 1 µM nisoldipine on APD alternans and restitution curve measured on multicellular tissue. **(a)** Illustrates representative original traces in control (red curve) and in the presence of 1 µM nisoldipine (green curve). Nisoldipine reduced the steady-state APD_90_ (bar graphs) measured at 500 ms BCL. **(b)** Represents a comparison between nisoldipine effect on restitution steepness (left y-axis) and on APD_25_ alternans (right y-axis) at a BCL of 500 ms. The nisoldipine reduced the slope of the restitution (green *versus* red curves) only in a slow phase, at DIs larger than 70 ms (significance marked with *). The APD_25_ alternans were significantly reduced at 250, 230, and 210 ms (green versus red symbols, significance marked with #). **(c)** Depicts APD_90_ restitution under control (red curve) and in the presence of 1 µM nisoldipine (green curve) at a BCL of 500 ms. However, the APD_90_ shortening after nisoldipine administration is clear, we can observe that the steepness of the curve primarily reduced at the slow phase.

In order to further investigate the role of I_CaL_ in the restitution, action potential measurements were performed in multicellular tissue. 1 µM nisoldipine was employed to inhibit selectively the I_CaL_ (Fig. 6). At first, the restitution protocol was applied at 500 ms of BCL then the alternans protocol was used from 250 to 190 ms BCLs. Administration of 1 µM nisoldipine suppressed the APD_90_ alternans at 250 ms of BCL (7.5 ± 1.2 ms *vs* 3.8 ± 0.8 ms; p < 0.05, n = 7, not shown in the figure). The APD_25_ alternans were reduced at 250 ms (14.7 ± 4.6 ms *vs* 4.4 ± 0.9 ms; p < 0.05, n = 7/7 hearts), at 230 ms (13 ± 3.1 ms *vs* 3.1 ± 1 ms; p < 0.05, n = 7), and at 210 ms (10.8 ± 2.6 ms *vs* 4.7 ± 0.7 ms; p < 0.05, n = 7, Fig. 6a,b). We found that 1 µM nisoldipine significantly shortened the baseline APD_90_ (187 ± 5 ms *vs* 159 ± 5 ms; p < 0.05, n = 7, bar graphs). In contrast, I_CaL_ inhibition failed to change the initial phase of the restitution steepness but changed it at DIs larger than 60 ms (Fig. 6b,c). Dynamic restitution was calculated from the APD alternans protocol and the effect of nisoldipine was investigated on the restitution slope. It was found that 1 µM nisoldipine does not alter the dynamic restitution slope (1.31 ± 0.1 *vs* 1.2 ± 0.1; n = 7; Figure not shown).

## Discussion

The major findings of this study are the followings: (1) in tissue AP measurements, the development of AP alternans could be evoked irrespective of the restitution slope, suggesting a minor fidelity of restitution curve in prediction of alternans. (2) In our experiments, CaT alternans were not observed in the absence of I_CaL_ alternans (3) I_CaL_ has little influence on the early phase of APD restitution curve.

### Has any role of restitution in the alternans development?

The general view is that membrane voltage-driven alternans are determined by a single parameter: APD restitution^5^. The restitution quantifies the relationship between APD and preceding DI. The major factors governing APD restitution are recovery from inactivation of the inward currents and deactivation of outward currents, as well as Ca^2+^ cycling also affects restitution through Ca^2+^ dependent currents^28^. Our results, similar to those that were reported in other previous studies^1,18^, do not support previous assumption by Nolasco and Dahlen suggesting that the steepness of restitution reflects the susceptibility of alternans^17^. The restitution hypothesis claims that alternans will occur when the restitution slope is larger than 1. In our tissue AP measurements, the restitution steepness did not influence the *development* of alternans (i.e. could be triggered regardless of the restitution steepness), rather the basic cycle length: alternans occurred in all cases (20/20 experiments) when the BCL was 250 ms or shorter. In our experiments, alternans *occurred* even if the restitution steepness (S1S2 or dynamic) was smaller than 1, however, the *extent* of alternans was weakly/moderately connected to restitution steepness. These results suggest that restitution steepness may have little fidelity in prediction of development of cardiac alternans. At the same time, it is important to note that several transmembrane ionic currents having potential role in alternans development do not contribute to the rapid restitution phase equally^29^.

The idea that alternans may have a restitution-independent cause has been recognized by Wu and Patwardhan. Using a feedback-based pacing protocol where diastolic intervals were selected explicitly and independently of APDs it was possible to investigate whether alternans occur when diastolic intervals preceding each AP do not change^30^. It was shown that identical diastolic intervals were also followed by APD alternans claiming that diastolic interval dependent restitution may not be directly linked to alternans development^30^.

### Do CaT alternans require action potential alternans?

Our current clamp experiments (Fig. 3) revealed a close relationship between APD and CaT alternans. This correlation implies that membrane potential or Ca^2+^ could be a potential driver of the alternans, however when membrane potential did not alternate the beat-to-beat CaT remained also unaltered.

A bidirectional coupling exists between AP and Ca^2+^-handling, and I_CaL_ is a major determinant of both actual APD and Ca^2+-^transients. When the Ca^2+^_i_ alternates, it significantly alters the APD via the balance of I_CaL_ and I_NCX_ causing positive or negative Ca^2+^ to Vm coupling^31^.

Several voltage-clamp studies reported that notable Ca^2+^ transient alternans were observed in the presence of non-alternating AP sequence^13–16^. These results were interpreted as the alternans were arose directly from the Ca^2+^ handling dynamics and not only the passive response of the beat-to-beat alternans of the AP^13^. In contrast, the data of this study indicate that membrane potential (i.e. AP alternans) is required for the development of CaT alternans in our experiments, and may support the important role of I_CaL_ and the bidirectional coupling between AP and Ca^2+^ handling in the development of alternans.

### I_CaL_ fluctuation is able to ignite CaT alternans

Previous results are controversial regarding the role of I_CaL_ in APD alternans initiation: the incomplete time-dependent recovery of I_CaL_ was found to cause alternans in several studies^8,21,32,33^. In a computer modelling study of ischaemic myocytes, alternans have been proposed to appear due to the interplay of I_CaL_ and potassium transient outward currents^34^. In our previous study we found that selective Na^+^/Ca^2+^ exchanger inhibition decreased alternans without influencing the restitution slope while indirectly inhibits I_CaL_^35^.

In contrast, several papers found in both atrial and ventricular myocytes that alternans can occur while peak I_CaL_ remained unchanged from beat to beat^14–16,36,37^. Additionally, numerous papers demonstrated mechanical and CaT alternans in the absence of AP alternans^2,13,14,16,37^.

In our experiments we found that oscillation of the I_CaL_ recovery time (that is often developed during alternans) is able to induce I_CaL_ peak fluctuation—as was expected, and the relationship between I_CaL_ and CaT amplitude alternans was closely coupled (Fig. 4). In line with previous results demonstrated in Fig. 3, the high pacing rate per se was insufficient again to induce CaT alternans, since when I_CaL_ alternans were small the corresponding CaT alternans were also restricted. The magnitude of I_CaL_ alternans was not altered even if the Ca^2+^_i_ was buffered (Supplementary Fig. S4) indicating the membrane potential origin of the I_CaL_ fluctuations.

At the same time, it is important to note that AP morphology and alternans influence the SR Ca^2+^ content and the efficiency of the excitation–contraction coupling^21^. Therefore, the actual SR Ca^2+^ content—together with I_CaL_—may have important contribution to the development of alternans^15^, however, this was out of the scope of this study.

### Does I_CaL_ contribute to alternans development in a restitution independent manner?

Since previous experiment showed that I_CaL_ can induce CaT alternans via alterations of the recovery period (i.e. voltage-dependent manner) the relationship of I_CaL_ with the restitution slope was investigated. Several papers reported that I_CaL_ is an important contributor of the restitution slope^38–40^.

In order to address this question the I_CaL_ kinetics was investigated under different recovery intervals. Our current measurement in the presence of unbuffered intracellular solution revealed that under short diastolic intervals—that corresponds to the fast phase of restitution—the recovered fraction of the I_CaL_ is relatively low (Fig. 5). Similarly, our tissue AP experiments with 1 µM nisoldipine (Fig. 6) demonstrated that selective I_CaL_ inhibition does not influence the fast phase of the restitution curve. This fact, in line with previous report^41^, may indicate that I_CaL_ has a minor role in shaping the fast phase of the restitution, presumably because highly incomplete recovery allows small I_CaL_ during short diastolic intervals. Furthermore, according to previous study^42^, the failure of nisoldipine to change restitution steepness could be also attributable to the relatively small effect on the baseline APD (~ 20%). Previous paper also reported no change in the time constant of electrical restitution of human ventricle after application of 1 µM nisoldipine in human^29^. At the same time, in line with our results, a numerical simulation demonstrated that I_CaL_ suppression reduces alternans^32^.

Previous studies reported that I_CaL_ blocker verapamil flattened the restitution curve^38,39^, however it is known that it also inhibits the I_Kr_ in submicromolar level^43,44^ that was not taken into account in those studies. I_Kr_ suppression was reported to flatten restitution^42^, while in contrast, dihydropyridines did not inhibit the I_Kr_^43,44^. Therefore, verapamil could not be considered as a suitable tool to assess the role of I_CaL_ in restitution, and its additional I_Kr_ suppressing effect makes the data interpretation difficult.

### Possible role of cardiac memory

Cardiac memory is a term introduced by Rosenbaum et al.^45^. The short term cardiac memory reflects the effect of pacing “history” on the APD, therefore, the relationship of alternans and APD restitution is complicated by the possible presence of the short-term cardiac memory. In our measurements the S1S2 restitutions measured at different BCLs and dynamic restitutions are different (Supplementary Fig. S3), indicating the presence of memory in the system. Several studies claimed that the presence of cardiac memory decreases the reliability of the restitution in prediction of alternans^46,47^. Therefore in our experiments the cardiac memory could be also an important contributor that reduces the coupling between alternans and restitution slope.

## Conclusion

In this study we found that I_CaL_ has minor contribution in shaping the fast phase of restitution curve. This suggest that I_CaL_—if it contributes to the mechanism of alternans—could be an additional factor that attenuates the reliability of APD restitution slope to predict alternans.

Since I_CaL_ can induce alternans in voltage-dependent manner (i.e. by the change of the recovery period) it could contribute to the development of a voltage-driven alternans that are largely independent of restitution.

## Limitations

The alternans-restitution comparison was investigated in endocardial tissue therefore it does not represent the whole heart. Other cell layers, such as midmyocardial cells or Purkinje fibers may exert different behavior.

## Supplementary Information

Supplementary Information.
