# Supplementary material for: The development of L-type Ca2+ current mediated alternans does not depend on the restitution slope in canine ventricular myocardium
Source: Sci Rep. 2021 Aug 17;11:16652. doi: 10.1038/s41598-021-95299-7 (PMC8371021; doi:10.1038/s41598-021-95299-7)
Supplement: Supplementary file 1 — Supplementary Information. [file 41598_2021_95299_MOESM1_ESM.docx]

**Supplementary Material**

**The development of L-type Ca^2+^ current mediated alternans does not depend on the restitution slope in canine ventricular myocardium**

Noémi Tóth^1^, Jozefina Szlovák^1^, Zsófia Kohajda^2^, Gergő Bitay^1^, Roland Veress^3^, Balázs Horváth^3,4^, Julius Gy. Papp^1,2^, András Varró^1, 2, 5^, Norbert Nagy^1, 2*^

^1^ Department of Pharmacology and Pharmacotherapy, Faculty of Medicine, University of Szeged, Hungary

^2^ ELKH-SZTE Research Group of Cardiovascular Pharmacology, Szeged, Hungary

^3^ Department of Physiology, Faculty of Medicine, University of Debrecen

^4^ Faculty of Pharmacy, University of Debrecen

^5^Department of Pharmacology and Pharmacotherapy, Interdisciplinary Excellence Centre, University of Szeged, Szeged, Hungary

**Running title:** Restitution-independent alternans and L-type Ca^2+^ current

^*^**Correspondence:**

Dr. Norbert Nagy

Department of Pharmacology & Pharmacotherapy

Faculty of Medicine, University of Szeged

H-6720 Szeged, Dóm tér 12, P.O. Box 427

Hungary

Tel: (36) (62) 545 682

Fax: (36) (62) 545 680

E-mail: nagy.norbert@med.u-szeged.hu

**Supplementary Figure S1**

**
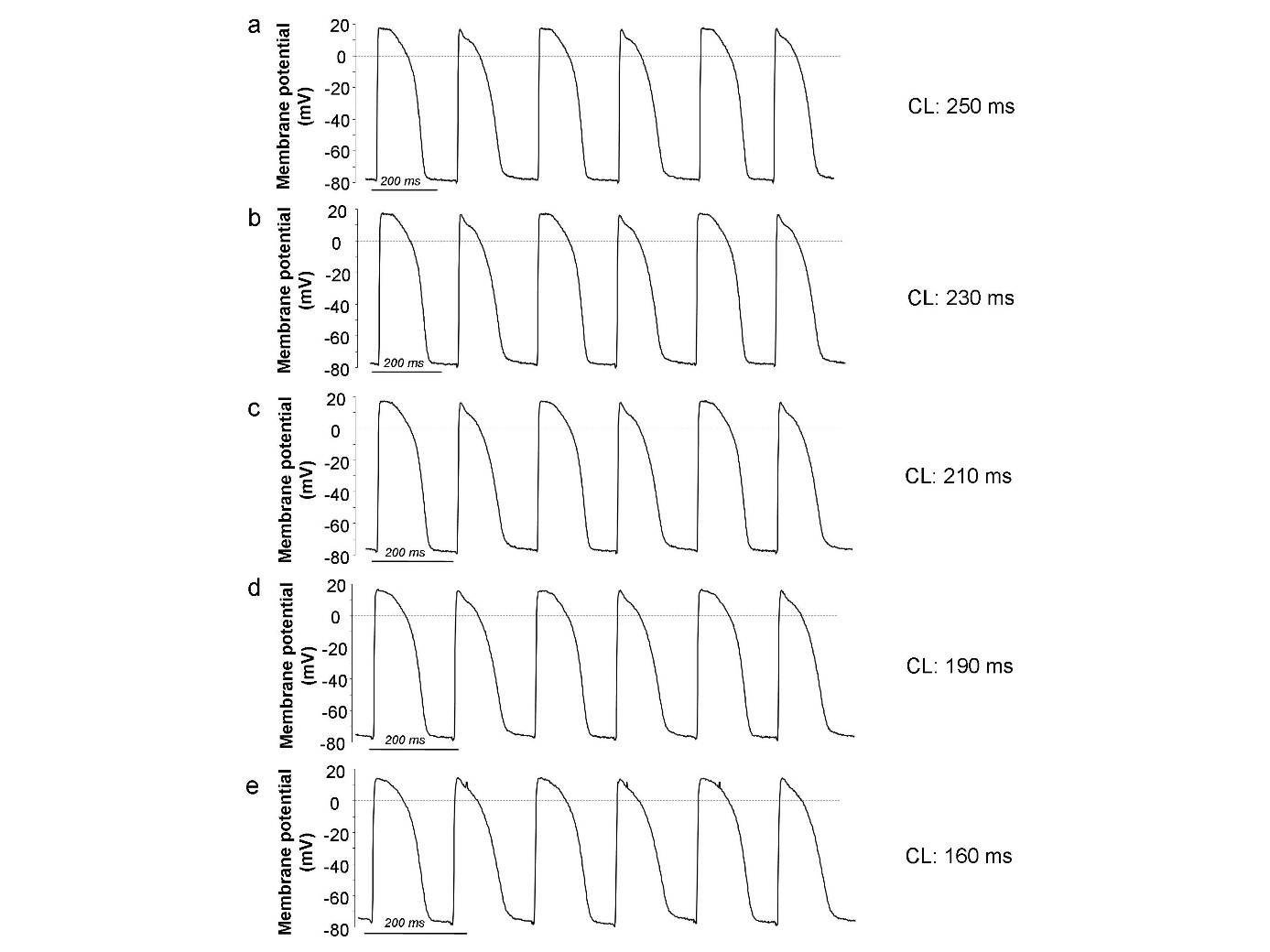
**

**Supplementary Figure S1: Representative original action potential traces at various cycle lengths.** Action potentials were measured by using pacing cycle lengths of 250 ms (**panel a**), 230 ms (**panel b**), 210 ms (**panel c**), 190 ms (**panel d**) and 160 ms (**panel e**).

**Supplementary Figure S2**


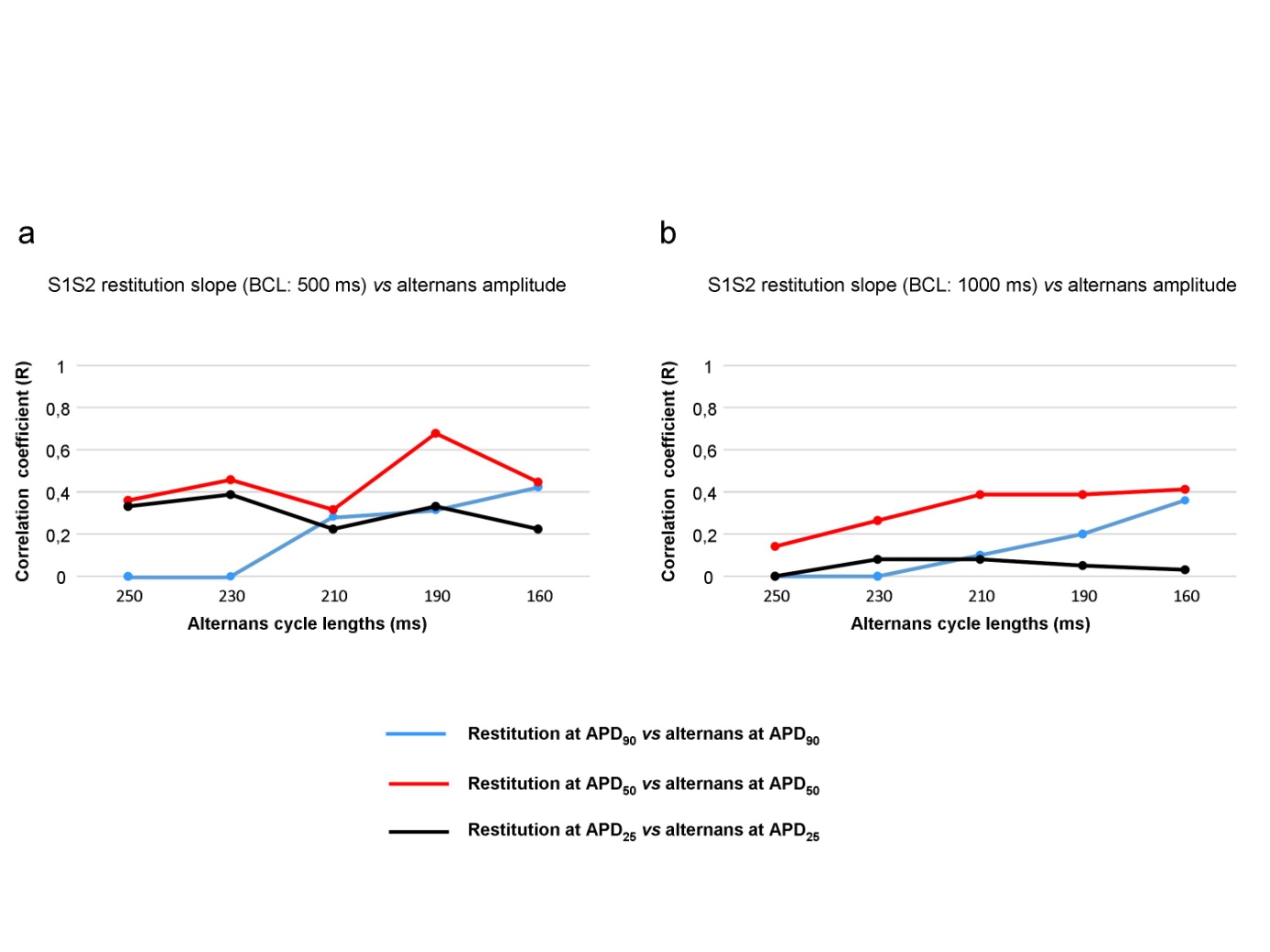


**Supplementary Figure S2: Correlations between S1S2 restitutions and alternans amplitudes at different cycle lengths.** S1S2 restitution was measured at BCL of 500 ms (**panel a**) and at BCL of 1000 ms (**panel b**). In both cases, blue lines represent correlation between restitution slope calculated at APD_90_ level and alternans at APD_90_ level. Red lines demonstrate correlation between restitution slope at APD_50_ level and alternans at APD_50_. Black lines show correlation between restitution slope at APD_25_ level compared to APD_25_ alternans.

**Supplementary Figure S3**


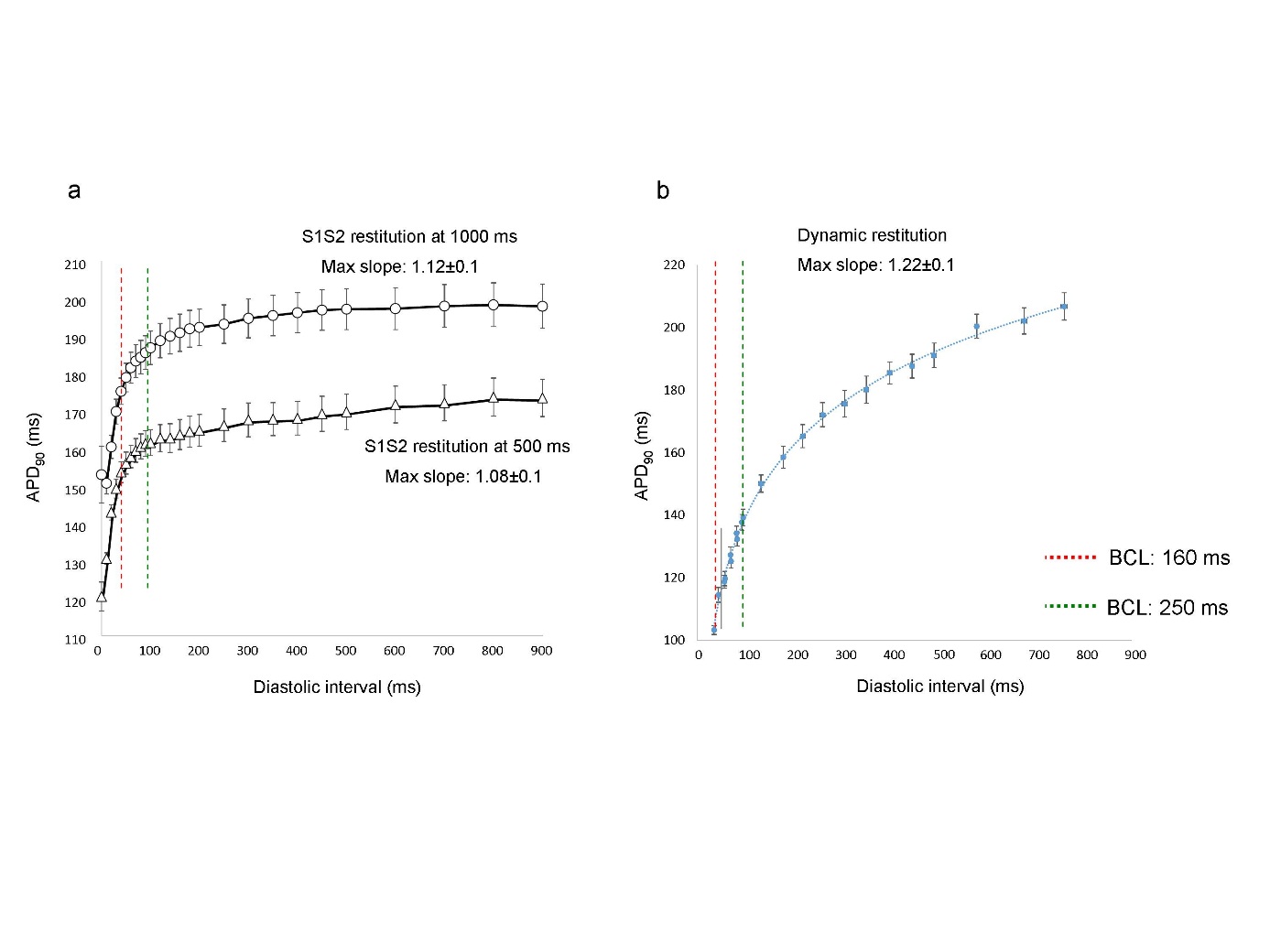


**Supplementary Figure S3**: **Relationship of restitution curves and diastolic intervals of alternans. Panel a** represents S1S2 restitutions measured at 500 ms (open triangle) and at 1000 ms (open circle). Vertical red dashed line indicates the relationship of S1S2 restitution slope and the shortest diastolic interval during alternans (BCL: 160 ms), vertical green dashed line indicate the longest diastolic interval during alternans (BCL: 250 ms). **Panel b** depicts the dynamic restitution curve. Average diastolic intervals of alternans at different cycle lengths are compared to the restitution slope. Vertical dashed red line indicates the shortest diastolic interval during alternans (BCL: 160 ms), vertical green dashed line show the longest diastolic interval during alternans (BCL: 250 ms). The restitution slope is >1 to the left of the vertical grey straight line, and <1 to the right of this line.

**Supplementary Figure S4**


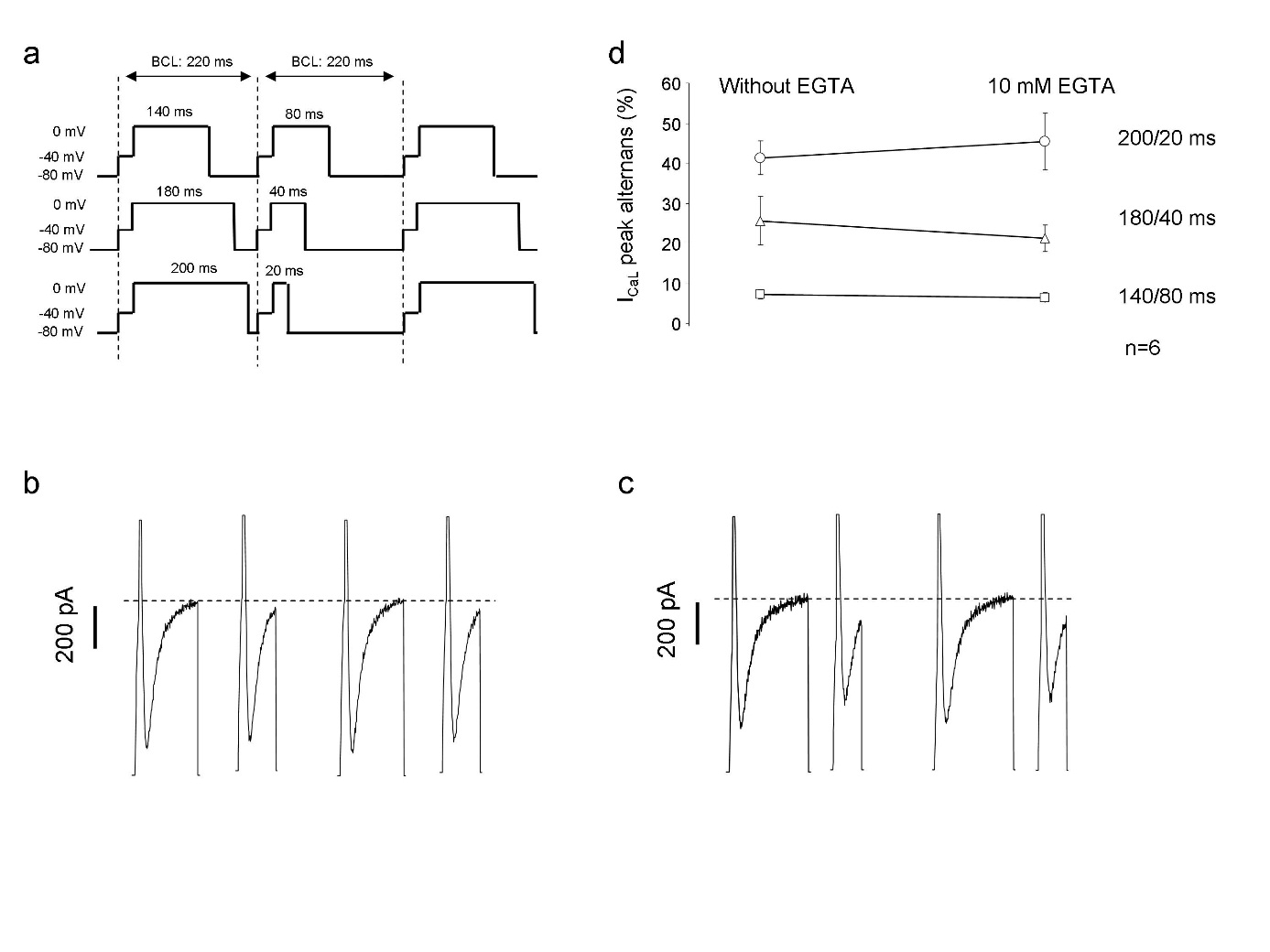


**Supplementary Figure S4**: **I_CaL_ in the presence of buffered intracellular milieu**. I_CaL_ were evoked by 3 subsequently applied alternating voltage clamp pulses, having a basic cycle length of 220 ms in all cases (**panel a**), identical to **Figure 3**. **Panel b** illustrates a membrane current evoked by a 140/80 ms voltage protocol. **Panel c** represents alternating I_CaL_ during application of 180/40 ms protocol. **Panel d** illustrates a comparison of I_CaL_ alternans in the absence and presence of EGTA. I_CaL_ peaks exerted identical magnitude of alternans during all protocols regardless of Ca^2+^ release.
